# IMPACT OF THE COVID-19 QUARANTINE ON THE MENTAL AND EMOTIONAL HEALTH OF POST-BARIATRIC SURGERY WOMEN: A QUALITATIVE STUDY

**DOI:** 10.1590/0102-6720202500009e1878

**Published:** 2025-04-14

**Authors:** Alisson Padilha de Lima, Isabela Gouveia Marques, Karla Fabiana Goessler, Roberto De Cleva, Marco Aurélio Santo, Hamilton Roschel, Bruno Gualano, Fabiana Braga Benatti

**Affiliations:** 1 Faculdade IELUSC, School of Physical Education – Joinville (SC), Brazil; 2 Universidade de São Paulo, Faculdade de Medicina, Hospital das Clínicas, Center of Lifestyle Medicine; Laboratory of Assessment and Conditioning in Rheumatology – São Paulo (SP), Brazil; 3 Universidade de São Paulo, Faculdade de Medicina, Escola de Educação Física e Desporto, Applied Physiology and Nutrition Research Group – São Paulo (SP), Brazil; 4 Universidade de São Paulo, Faculdade de Medicina, Department of Gastroenterology – São Paulo (SP), Brazil; 5 Universidade Estadual de Campinas, Faculdade de Ciências Aplicadas – Limeira (SP), Brazil.

**Keywords:** Bariatric Surgery, COVID-19, Mental Health, Women's Health, Cirurgia Bariátrica, COVID-19, Saúde Mental, Saúde da Mulher

## Abstract

**BACKGROUND:**

The coronavirus disease 2019 (COVID-19) greatly impacted patients undergoing bariatric surgery due to prolonged quarantine and lockdown measures.

**AIMS:**

The aim of this study was to qualitatively investigate the impact of the COVID-19 quarantine and lockdown measures on the mental and emotional health of post-bariatric surgery women.

**METHODS:**

A qualitative study was carried out, with individual interviews conducted via video calls using a video-communication service (Google Meet^®^). The moderator guide inquired about three pre-established topics based on the literature: mental and emotional health, social relationship, and the use of health technology.

**RESULTS:**

A total of 12 women participated in this study, with an average age of 43±9.83 years, a body mass of 82.33±13.83 kg, a height of 1.62±0.06 m, a body mass index of 26.32±2.97 kg/m^2^, and post-surgery time of 12.83±4.37 months. The interviews had an average duration of 50.71±7.26 min. Our results suggested a negative impact of the COVID-19 pandemic on aspects of mental and emotional health, such as increased anxiety, depressive symptoms, fear, stress, and anguish, which were somehow diminished in patients who were closer to family members. Bariatric surgery was mentioned as a positive aspect by the patients for coping with clinical risk conditions.

**CONCLUSIONS:**

The study showed a negative impact of the COVID-19 pandemic on aspects of mental and emotional health mostly due to lockdown measures, which led to social isolation and an increased burden with household chores.

## RESUMO

**RACIONAL:**

A doença Coronavírus 2019 (COVID-19) impactou fortemente os pacientes submetidos à cirurgia bariátrica devido às medidas prolongadas de quarentena e bloqueio.

**OBJETIVOS:**

Este estudo teve como objetivo investigar qualitativamente o impacto das medidas de quarentena e bloqueio da COVID-19 na saúde mental e emocional de mulheres pós-cirurgia bariátrica.

**MÉTODOS:**

Foi realizado um estudo qualitativo, com entrevistas individuais realizadas por meio de chamada de vídeo, utilizando um serviço de vídeo comunicação (Google Meet^®^). Um guia moderador com perguntas sobre três temas pré-estabelecidos com base na literatura foi realizado: saúde mental e emocional; relacionamento social; e uso de tecnologia em saúde.

**RESULTADOS:**

Participaram deste estudo 12 mulheres, com idade média de 43±9,83 anos, massa corporal de 82,33±13,83 quilogramas, altura de 1,62±0,06 metros, índice de massa corporal de 26,32±2,97 kg/m^2^ e tempo pós-operatório de 12,83±4,37 meses. As entrevistas tiveram duração média de 50,71±7,26 minutos. Nossos resultados sugeriram um impacto negativo da pandemia de COVID-19 em aspectos da saúde mental e emocional, como aumento da ansiedade, sintomas depressivos, medo, estresse e angústia, que foram de alguma forma diminuídos em pacientes mais próximos de familiares. A cirurgia bariátrica foi citada como aspecto positivo pelos pacientes para o enfrentamento das condições clínicas de risco.

**CONCLUSÕES:**

O estudo mostrou um impacto negativo da pandemia de COVID-19 em aspectos da saúde mental e emocional, principalmente devido a medidas de confinamento que levaram ao isolamento social e a um aumento da carga de tarefas domésticas.

## INTRODUCTION

Bariatric surgery is the cornerstone treatment for severe obesity^12^, with a high success rate of long-term weight loss and substantial improvements in metabolic health^8,32,35^. However, adequate post-surgery follow-up is crucial to help patients achieve the estimated weight loss and improvements in comorbidities^36^, in addition to preventing potential psychological disorders that may arise following surgery^13^.

The coronavirus disease 2019 (COVID-19) also had profound impacts on patients undergoing bariatric surgery, particularly due to the limited access to health care in the face of the prolonged quarantine and lockdown measures necessary to contain the virus spread. This has resulted in detrimental effects on long-term postoperative health, such as deterioration of long-term outcomes and lower weight losses^36^.

Although social distancing measures were effective in preventing COVID-19 infection, mobility restrictions, lack of social interaction, and limitation of daily activities impacted individuals’ mental health, generating frustration, feelings of insecurity, confusion, emotional isolation, and stigma^6,20,28^. Indeed, several quantitative studies have demonstrated various psychological symptoms arising from quarantine^8,30^, such as emotional imbalance^16^, depression^10^, stress^14^, lack of mood^21^, irritability^21^, insomnia^21^, post-traumatic stress symptoms^21^, anger^23,29^, and emotional exhaustion in working conditions^24^. Likewise, qualitative studies have found other psychological responses from the quarantine, such as confusion^5,10^, fear^9,14^, anger^10^, pain^9^, numbness^37^, and anxiety-induced insomnia^14^.

These effects have been particularly detrimental to people with obesity, with reports of increased anxiety and depression due to social isolation measures^22^. In addition to the psychological aspects that affect mental health during the quarantine and that may persist thereafter, some specific stressors to this population can include longer confinement, inadequate supplies, difficulty in obtaining health care, and financial difficulties^6^. Moreover, women, who are more commonly responsible for the bulk of domestic tasks^3^, may have been more impacted.

Thus, it is possible that COVID-19 and lockdown measures have highly impacted the mental health of women undergoing bariatric surgery. As they may face a heavier burden in dealing with the pandemic, it becomes fundamental to identify, understand, and advocate for their mental health since some of the pandemic-associated negative impacts on well-being and overall health may carry over in the long run. In this context, this study aimed to qualitatively investigate the impact of the COVID-19 quarantine and lockdown measures on the mental and emotional health of post-bariatric surgery women.

## METHODS

This study utilized the Consolidated Criteria for Reporting Qualitative Research (COREQ)^33^. This study is part of a larger randomized clinical trial aimed at gathering knowledge about the potential benefits of physical exercise in patients undergoing bariatric surgery (NCT02441361). Data reported herein were collected during the pandemic, and patients digitally provided their informed consent.

### Participants

The participants in this study were recruited from the Gastroenterology Department, Digestive Surgery Division of the Hospital de Clínicas. A convenience sample of women who underwent bariatric surgery was selected. The inclusion criteria were female patients, aged 20–60 years, those who underwent bariatric surgery (Roux-en-Y gastric bypass and sleeve gastrectomy) in the past 12 months, and those who did not participate in any exercise training protocol during the period of quarantine of the COVID-19 pandemic lockdown in São Paulo, from March 2020 to May 2020.

**Figure 1:**
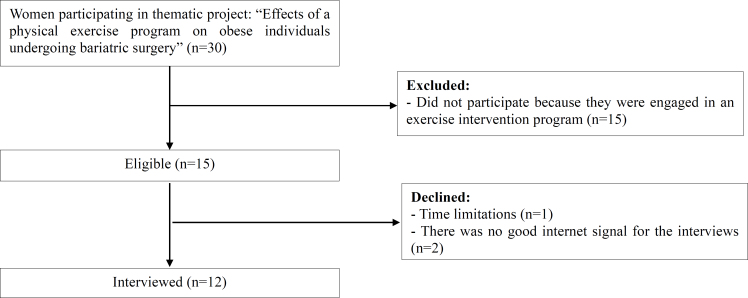
Flowchart of the recruitment process.

We emphasize that the exclusion criterion adopted for patients who participated in the physical exercise program was that these patients were part of the clinical trial and received follow-up and intervention for 6 months, while patients in the control group only had follow-up. Patients with a post-operative period exceeding 12 months, internet access limitations, and mental disorders that prevented the interview from being carried out were not eligible, as depicted in Figure 1.

### Research instruments

A guiding script was used to conduct individual interviews for data collection. The interviews were conducted by male postgraduate students and female postgraduate students, both researchers, with previous experience in qualitative studies.

A video call was scheduled using a video-communication service (Google Meet^®^) on the participants’ preferred day and time, with participants being at home. A pilot test was conducted before the beginning of the research with two patients, who did not participate in the protocol, to adjust the instructions and questions of the study. The moderator guide inquired about three pre-established topics based on the current literature: *mental and emotional health, social relationship,* and *the use of health technology*.

The "mental and emotional health" and "social relationship" topics were pre-established according to previously published studies that demonstrated the impact of the COVID-19 pandemic on patients who had undergone bariatric surgery, and the consequent restricted access to health care (including medical, nutritional, and psychological consultations) during the quarantine period, which could be harmful to long-term postoperative health^20,28,30,36^. Due to its relevance during the pandemic, we added the topic "use of technology for health care," as it yielded online consultations with specialists and telemedicine strategies to reduce the negative effects of the pandemic on health^15,22,36^.

In addition to the pre-established script as a guide, the moderators used probing questions that inquired participants about the impact COVID-19 had on their mental and emotional health, as summarized in Table 1.

**Table 1:** Interview guide

**1.**	Demographic and clinical characteristics Age, body mass, height, body mass index, race, level of education, occupation, marital status, residence, surgery time, and type of bariatric surgery
**2.**	Mental and emotional health – Positive probes: Tranquility, pleasure to stay at home, leisure time – Negative probes: Stress, anxiety, depression, anguish, fear, insecurity
**3.**	Social relationship – Positive probes: More time with family, time to talk with people who are far away – Negative probes: Family friction, arguments, division of domestic tasks, social distancing
**4.**	Use of health technology – Positive probes: Ease of access to the team of health professionals, freedom of choice of location and time for physical training, greater independence to become physically active and maintain regular training, feeling pleasure (motivation) with the training, feeling able to take care of your health – Negative probes: Lack of financial resources to access the Internet, difficulty handling mobile devices, difficulty understanding what needs to be done, fear of causing injury

Source: The authors.

### Data analysis

The interviews were transcribed *verbatim* in Portuguese and translated to English by bilingual and bicultural researchers. Thematic analysis was conducted by following three steps according to previous literature^34^.

1. Pre-analysis: Organization of the material in order to prepare it for the identification of the central idea. Pre-analysis was configured as the data organization phase itself, characterized as a moment of researcher's intuition, aimed to operationalize and systematize the initial ideas of data collection, culminating in an analysis plan. The pre-analysis phase consisted of three missions: the choice of documents to be analyzed; the formulation of hypotheses and objectives; and the construction of indicators that will support the final interpretation. These aspects of the first phase of analysis are interconnected but may not follow a chronological order^34^.
2. Exploration of the material: In-depth study of the data, aiming to identify the nuclei of meaning. After the pre-analysis phase and its different stages were satisfactorily completed, the material exploration phase was constituted as the systematic application of the decisions made, configuring itself in coding, decomposition, or enumeration operation, related to previously formulated rules^34^.
3. Treatment of the obtained data and interpretation: Regrouping of the data with the similarity of their meanings and the discussion in light of the constructed framework^34^. For coding and content analysis, the software NVivo^®^ version 11 was used.

In addition to the thematic analysis, we used word cloud analysis to shed light on the themes most addressed by the participants during the interviews. The figures were created using the Pro Word Cloud tool found in the office package Word 2016^®^.

### Ethical aspects

This research involved human beings, and all procedures carried out here respected the ethical principles described in the 1964 Declaration of Helsinki and its current amendments. The project was approved by the Ethics and Research Committee of the University Hospital, School of Medicine, Universidade de São Paulo (number: 4.294.103). Informed consent was obtained digitally (via Google Forms^®^) from all participants included in the study.

## RESULTS

A total of 12 women participated in this study, with an average age of 43±9.83 years, a body mass of 82.33±13.83 kg, a height of 1.62±0.06 m, a body mass index of 26.32±2.97 kg/m^2^, and surgery time of 12.83±4.37 months. Interviews averaged 50.71±7.26 min of duration. Other characteristics of the sample are shown in Table 2.

**Table 2:** Characteristics of the sample

Participants	Race	Marital status	Level of education	Employment status	Household members	Type of surgery
BAR 1	Black	Single	High school	Self-employed	OFMs	RYGB
BAR 2	Brown	Married	High school	Homemaker	Husband/sons	RYGB
BAR 3	Brown	Single	High school	Self-employed	OFMs	RYGB
BAR 4	White	Married	High school	Employed	Husband/sons	RYGB
BAR 5	White	Married	High school	Employed	Husband/sons	RYGB
BAR 6	White	Married	Higher education	Employed	Husband/sons	RYGB
BAR 7	Brown	Married	Higher education	Employed	Husband/sons	SG
BAR 8	Black	Single	Higher education	Employed	OFMs	RYGB
BAR 9	Brown	Widow	High school	Employed	OFMs	RYGB
BAR 10	White	Married	Higher education	Employed	Husband/sons	SG
BAR 11	White	Married	High school	Employed	Husband/sons	RYGB
BAR 12	Brown	Single	Higher education	Self-employed	OFMs	RYGB

OFMs: Other family members; RYGB: Roux-en-Y gastric bypass; SG: Sleeve gastrectomy.

**Figure 2:**
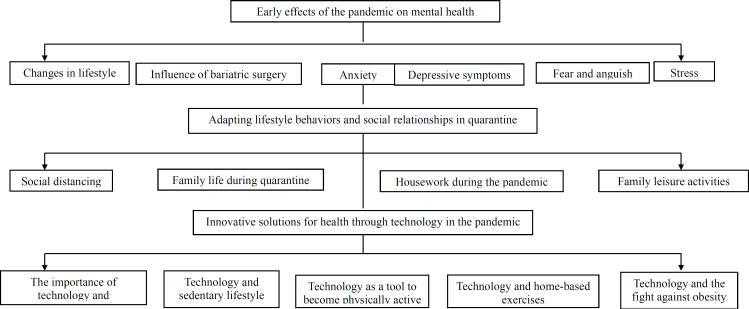
Flowchart resulting from the thematic analysis.

After thematic analysis of the content of the participants’ speech, the results were organized into topics and subtopics, as depicted in Figure 2.

### Early effects of the pandemic on mental health

#### Anxiety

Anxiety was one of the main aspects that influenced the mental health of bariatric patients. Notably, patients who were able to stay with their families had more control over their symptoms.

There was no tranquility at all; in the beginning of the pandemic, I felt more anxious and worried; I feel calmer now but there is no such thing as "tranquility" […] (BAR 1)

I even had to take some medication to decrease my anxiety […] (BAR 6)

I felt no impact because my family stayed together; I did not have any anxiety; after I had surgery, I felt calm, even during lockdown […] (BAR 10)

I had a lot of anxiety and lost a lot of weight, but it got better when later I was able to be closer to my family […] (BAR 3)

#### Depressive symptoms

Depressive symptoms were present in some bariatric patients, due to some factors such as staying at home for long periods and deprivation of contact with people, especially family members and friends.

There were moments when I got depressed, felt sad, wanting to cry for no specific reason […] (BAR 1)

[…] because I stay at home a lot, I feel depressed […] (BAR 4)

I had depressive symptoms and was distressed because some friends who worked at the bank were contaminated. I was sad […] (BAR 11)

I felt very impacted emotionally because we could not see our family members, so I got very depressed […] (BAR 12)

#### Feelings of fear and anguish during the pandemic

Fear and anguish were emotions reported by all patients, mainly due to the negative consequences that COVID-19 could cause before and after the surgery, the fear of contaminating family members, in addition to the isolation from friends and family members.

[…] We did not have peace of mind because we felt that despair from the grocery shopping that we had to wash and sanitize. We had to be very cautious when we got home because my mother is elderly, so we were traumatized and afraid of contaminating ourselves and transmitting it to others. (BAR 2)

My biggest fear was contaminating my daughter. But I was also afraid for myself because they said that my immunity would be lower because I had the surgery. […] I felt very insecure, and the anguish of being away from the family, not being able to travel, not being able to go out, and not being able to do anything generated more anguish. (BAR 3)

You normally feel a lot of anguish after bariatric surgery and, when this virus came up, this feeling increased […] (BAR 6)

At first, I was worried because my mother and father are elderly and I was afraid of contaminating them. But after a few days, I calmed down […] (BAR 11)

For some patients, having chronic diseases before bariatric surgery made them consider themselves at risk during this pandemic period.

The fear and anguish were very high, especially when my brother-in-law got COVID-19. I am in the risk group for having high blood pressure, diabetes, and asthma. Furthermore, my whole family is also in the risk group […] (BAR 6)

#### Quarantine stress

Spending more time at home was one of the biggest challenges for bariatric patients. In addition, external factors such as the interrupted work routine for some patients and the intensified work routine for others, as well as the increased time spent with family, favored the increase in stress during this period, as well as an increased time spent with family favored the increase in stress during this period.

[…] We spent more time at home, and with the new family routine, stress levels increased. Men are more stressed indoors, so my husband stayed a little longer, which made me nervous too. (BAR 5)

In the first few months, there was more stress. As we stayed together at home, there was family stress […] (BAR 10)

I am a health professional; at first, I spent practically the entire shift inside the operating room, and with that rush, we were very stressed. (BAR 12)

#### Influence of bariatric surgery in coping with the pandemic

Many patients reported the importance of bariatric surgery as a treatment modality and preventive measure of health problems, as it favors weight loss and reduces the symptoms of chronic diseases. Moreover, other precautions, such as the intake of vitamins associated with surgery, were also considered as a preventive factor for coping with the COVID-19 pandemic.

If it was not for my bariatric surgery, I would not be here doing this interview. Because my previous weight was 134 kilos and I had asthma, I would probably not resist this virus. (BAR 2)

[…] After the bariatric surgery I was feeling stronger to face the pandemic […] (BAR 9)

[…] After the bariatric surgery I was calm, even in the quarantine period […] (BAR 10)

[…] Because of the vitamins we take after bariatric surgery, I feel I have a good immune system […] (BAR 12)

#### Impact of lockdown on lifestyle and mental health

Patients highlighted that the lack of physical activity and outdoor leisure activities and increased sedentary behavior were factors that impacted mental health during quarantine for bariatric patients.

It would be necessary to go to the gym, dance, and go out. These changes could improve my mental and physical health, but now we must let go of that because of the pandemic because I do not feel safe yet. (BAR 1)

[…] I became very sedentary because I couldn't even leave the house, and on my street, some people had COVID […] (BAR 4)

Patients also mentioned that the lack of psychological follow-up, the possibility of getting vaccinated and, thus, returning to their normal daily routine impacted their mental health.

For those of us who underwent bariatric surgery, to have good mental health during this pandemic, we would have to have the psychological follow-up and talk to other people who had the surgery and who were losing weight to encourage us to follow the same healthcare […] (BAR 5) To improve my mental health, we would have to get vaccinated soon […] (BAR 8)

**Figure 3:**
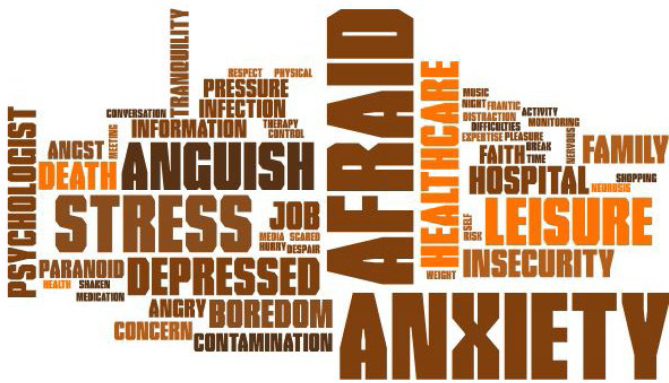
Mental and emotional health word cloud.

Based on the thematic analysis of the interviews, we highlighted the themes influencing mental health that most appeared during the patients’ speech. For this purpose, we used the word cloud, which organizes the themes from largest to smallest, based on the frequency of appearance during the speeches, as depicted in Figure 3.

### Adapting lifestyle behaviors and social relationships in quarantine

#### Social distancing

Social distancing was one of the main challenges facing the pandemic, and bariatric patients did it very carefully. Also, some preventive measures were mentioned during the quarantine period to minimize the risk of contamination, such as hand sanitizing and the use of face masks.

We followed care and social distancing, used alcohol and masks, and washed our hands, that is what we were able to do at that moment. (BAR 6)

We respect the social distance, do not receive anyone at home, we are more isolated, and there was not even a visit from family members. (BAR 9)

On the other hand, the restriction of family contact and get-togethers was an impacting factor during the quarantine period. This restriction was due to the advanced age of the grandparents and to prevent the contamination of other family members (OFMs).

We respect social isolation a lot, we stayed like this for almost three months, only I would leave the house to buy food and then come back because my grandmother is ninety years old and needed to be very careful. (BAR 10) We fully respect social distancing, leaving the house only to go to the market, and my father had this task. (BAR 11)

#### Family life during quarantine

To some patients, quarantine did provide rescue and the opportunity to spend more time with children, spouses, and OFMs.

It was great to spend more time with the family. I have nothing to complain about […] (BAR 3)

We learn in coexistence and with conflicts, to respect people's individuality more, because we normally don't spend so much time with the family, we spend more time at work. (BAR 5)

It was very nice to spend more time with my son, he used to complain that I worked a lot. I was now able to pay more attention and stay at home more. (BAR 7)

#### Housework during the pandemic

The division of household tasks was a conflicting aspect during the pandemic, as patients oversaw most of the household tasks. Although husbands and children took turns for a few moments to contribute to the organization of the house and avoid family conflicts during this period of isolation, patients were the most burdened.

[…] it was more difficult to share some household chores, but everyone helped with the tasks such as cleaning the house and washing the dishes. (BAR 2)

I always do the household chores; the only help was with taking care of the baby (daughter). (BAR 3)

The household tasks were almost entirely done by me, like washing the dishes, washing clothes, cooking, going up and down stairs, and only the cleaning was done by my daughter. (BAR 4)

#### Family leisure activities

During this period of quarantine, few patients had the opportunity to perform leisure activities. Two patients reported that they conducted recreational activities at home, such as board and active games.

We invented some leisure activities to do at home, like board games. (BAR 1)

[…] we play "monopoly," do pillow fights, and watch movies. (BAR 2)

**Figure 4:**
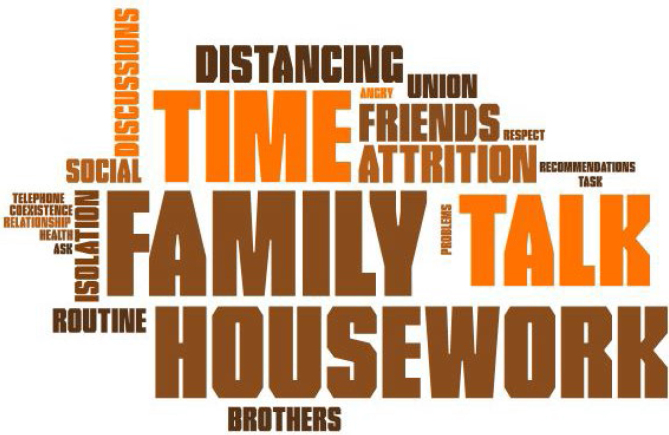
Social relationship word cloud.

Figure 4 presents the most discussed topics in interviews with women undergoing bariatric surgery. For the subtopic adapting lifestyle behaviors and social relationships during quarantine, topics such as family, friends, speech, and time were highlighted.

### Innovative solutions for health through technology in the pandemic

#### The importance of technology and telemedicine for health care in quarantine

Telemedicine was a very effective quarantine tool for the treatment and health care of patients who had had bariatric surgery. This support was essential in terms of treatment and guidance after bariatric surgery.

I had several medical consultations via WhatsApp, which was very important. (BAR 1)

I think telemedicine can be good, over the phone the simplest needs can be addressed. Although, if the health situation requires more detailed care, it has to be in person, it needs medical contact and physical exams. (BAR 5)

I think we can take care of health through technology. I like to research and use apps that inform us about the questions we have about health. (BAR 6)

I would like to have telemedicine with the Rheumatologist to take better care of my health. (BAR 9)

The use of technology favored the mental health care of many bariatric patients during quarantine. In addition, opportunities for relaxation and entertainment helped overcome this moment of social isolation.

Technology was very good in quarantine, it saved a lot of people. If we did not have the technology, I think a lot of people would have been in a worse mental health situation. (BAR 10)

Technological advances related to surgical techniques were topics mentioned by the patients.

Technology favors us a lot these days, surgeries are a great example of this technological advance, made by video, several surgeries are not as invasive as they used to be with large cuts. An example of this advance is our bariatric surgery. (BAR 12)

#### Technology and sedentary behavior

Sedentary behavior was an aspect that increased during this period of isolation at home. Many patients reported that staying at home and spending more hours using technology favored this increase in a sedentary lifestyle.

Unfortunately, people are overusing technology and increasingly becoming sedentary. There is no more interaction and contact with other people, as in the past. (BAR 1)

The fact that staying at home for a long time made us more sedentary, we spent most of the time sitting and watching television. (BAR 2)

I believe it is a possibility to do physical activity because sometimes we can't pay a professional to do exercise, and we get sedentary, and we get old, our body is a machine if you don't take care of it, it will rust. (BAR 6)

#### Technology and obesity

Another topic mentioned by bariatric patients was the increase in obesity and its association with the misuse of technology. However, the ease of buying and ordering fast food through apps, increased screen time (computer, cell phone, television), and physical inactivity factors contribute to this deleterious effect on technology-related obesity.

I think technology can increase obesity, because people spend a lot of time sitting and eating a lot of fast foods, in addition to spending a lot of hours on the internet. (BAR 2) For me, obesity can be a person's genetics or if they tend to put on weight because there are people who spend all day at the computer and with a lot of food and don't get fat, so I think it has nothing to do with technology. (BAR 3) It can help in my health, in the fight against obesity, so that people don't stand still and avoid more health problems. (BAR 5)

Technology favors and at the same time, it has disadvantages, because people do not know how to use it correctly. Even in children, who no longer play outside as they used to, it contributes to the increase in obesity. (BAR 6) I believe it can influence obesity, because people use their cell phones and television a lot, in addition to sitting and eating all the time, which will favor weight gain. (BAR 7) Currently, we have a lot of diet and care information on the internet, which can help in the fight against obesity. (BAR 11)

Technology can be used to favor and harm health. As an example, in this quarantine people watched more television and videos, and spent more time sitting. On the other hand, many people posted gymnastics videos and moved more, favoring weight loss. (BAR 12)

#### Technology as a tool for adherence to physical activity

Social media, videos, apps, and information on the Internet may favor the practice of physical activity. Technology may provide effective tools to promote physical activity, as highlighted by the patients.

Technology favored patients who had bariatric surgery, not only to exercise, but also to help them communicate with relatives who were far away, with family, and with friends, in this moment of isolation. (BAR 2)

The technology could help me become more active, because I can't maintain regularity of training due to schedules, I get home late and I have to do the housework and take care of my daughter. So I want to go to the gym in person, but I don't have time. (BAR 3)

The ease of training with the help of technology is good because you can choose the best times, and it doesn't interfere with your other activities during the day, which helps me to stay active. (BAR 5)

It would be great for me to do exercises through technology, to be able to choose a time, and then I would commit, because without the commitment I know I won't do it. That way, it could help me become physically active because I know how much time I have and the commitment to wake up at that time. (BAR 7)

Through technology, we can become more physically active and take better care of our health as well. (BAR 12)

#### Technology, motivation, and supervision professionals for the practice of home-based exercises

With the restrictions imposed by the COVID-19 pandemic, places for physical exercising, such as public squares and gyms, were impossible to utilize. In this context, a viable and effective alternative was the use of technology to perform supervised home-based exercises.

As the gym closed and I was undergoing a weight loss procedure, I couldn't stand still. I started doing exercises at home, like Zumba, using technology. (BAR 2)

I am not motivated to do online exercises alone; however, if it were on a video call, I would feel more motivated because someone would be looking at me, talking to me, and motivating me. Moreover, having the supervision of a Physical Education professional would make me feel safer. (BAR 3)

I really like to use technology to practice home-based exercises. My daughter and I exercise online whenever possible. I would like to practice home-based exercises under the online supervision of a professional. (BAR 5) It is viable to exercise at home. Technology can make it easier, but you need a professional to guide you because sometimes you can feel some pain during the exercise and if you are being accompanied by a professional you will know what to do at that moment. (BAR 6)

I enjoyed doing home-based exercise through an app I downloaded; my leg was no longer sore; the exercise was effective. But I prefer to go to the gym because I can have the supervision of a professional and other people exercising. I don't have the discipline to train alone. (BAR 8)

Training at home would be easier, being able to choose the time, I think I could become more physically active as well, I would feel motivated, and it could help with my physical and mental health. (BAR 9)

It would be great to have a professional encouraging you and showing you how to exercise because it's not the same thing to train alone. (BAR 10)

**Figure 5:**
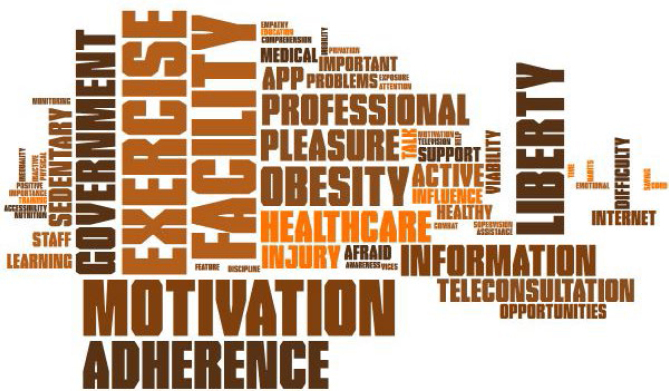
Health technology word cloud.

The word cloud in Figure 5 demonstrates that technology may be relevant to improve physical activity practices at home and to post-surgery health-care follow-up.

## DISCUSSION

To the best of our knowledge, this is the first study to qualitatively analyze the impact of COVID-19 and lockdown measures on the mental and emotional health of post-bariatric patients. Our results suggested a negative impact of the COVID-19 pandemic on aspects of mental and emotional health, such as increased anxiety, depressive symptoms, fear, stress, and anguish. Bariatric surgery was, however, a positive aspect mentioned by the patients for coping with clinical risk conditions, as obesity was reported as a high-risk factor for mortality from COVID-19 since the very beginning of the pandemic. Moreover, being able to spend more time with the family and carrying out leisure activities were highlighted as positive aspects, whereas social distancing and the division of household chores were reported as negative aspects. In addition, our patients reported that technology was an innovative solution for health care through the delivered information and telemedicine, thus helping with post-surgery follow-up treatment. It also served as a potential tool for a more active lifestyle as well as an instrument for home-based exercising. However, they reported that excessive time spent using technology could be negatively associated with an increased sedentary lifestyle and obesity^25^.

The negative feelings reported by patients, such as increased anxiety, depressive symptoms, fear, stress, and anguish, had a significant impact on the mental and emotional health of bariatric patients^16^. These results corroborate the findings reported in the general population^2,7^ and those of Walędziak et al.^36^, who specifically reported a high level of anxiety in post-bariatric surgery patients. In addition, limited access to medical and health care resulting from the quarantine lockdown resulted in a significant increase in anxiety levels and a deterioration in bariatric surgery outcomes, such as weight loss^4,16^.

In our study, the uncertainty of the consequences of COVID-19 after the surgery was mentioned as a contributing factor by some patients, as they were told that their immunity could be impaired. Moreover, lockdown measures and deprivation of contact with friends and family members also played a major role. Notably, being close to family members had opposite effects: although it minimized feelings of anxiety in some patients, it increased the feeling of fear and anguish in others due to the fear of contaminating them. This was probably a relevant factor in this study because three of the patients were health professionals working at the frontline.

Obesity was, since the very beginning of the pandemic, associated with an increased risk of the disease course of COVID-19^1,38^. As such, many patients appreciated the importance of bariatric surgery and the associated weight loss with the prevention of potential disease aggravation in the case of COVID-19 infection. Interestingly, despite the lack of scientific evidence, others believed that the intake of vitamins associated with the surgery was also a preventive factor for coping with the COVID-19 pandemic.

Lockdown measures were one of the biggest social and emotional challenges for the population in general^4,27^, which can be extended to our sample of bariatric patients. They reported having been very careful in maintaining social distancing and following sanitary recommendations. Notably, to some patients, the lockdown provided an opportunity to spend more time with children, spouses, and OFMs. However, to other patients, the excessive time spent together was regarded as negative and stressful. As expected, as our sample was composed of the most burdened by them, which was probably a contributing factor to the increased number of women, many of the patients oversaw household tasks^18^ and, thus, were stress reported.

Many patients reported that psychological follow-up could have been an important tool in helping cope with the pandemic. Although this was not available to most of our sample, many patients mentioned that the use of technology favored their mental health care during quarantine, as it helped overcome social isolation and provided opportunities for entertainment and relaxing moments^19^. Moreover, they mentioned that the use of technology, particularly telemedicine, was an effective tool for their follow-up treatment and guidance after the surgery. It is noteworthy that not all patients had access to this tool. Possibly, the low socioeconomic status of our samples and lack of computer knowledge were barriers to the effective implementation of this health resource. Indeed, an Italian study by Runfola et al.^31^ argues that to implement technology and telemedicine in public health with quality, these patient-related barriers need to be overcome for effective integration into outpatient practice.

Technology was also mentioned by the patients as having opposite effects on lifestyle. Many patients reported that staying at home and spending more hours using technology increased time spent on screen and, thus, favored a sedentary lifestyle, as well as a less healthy diet, due to the ease of buying and ordering fast food through apps. We have recently shown that post-bariatric patients had worsened quality of life and increased physical inactivity during the pandemic^17^. On the other hand, a few patients recognized the increased availability of videos on social media and apps incentivized the practice of home-based exercises, which could actually reduce sedentary behavior and improve the functionality of post-bariatric patients. Home-based exercise has emerged as an effective alternative to increase physical activity levels during this period of isolation^11^. Recently, we demonstrated that a 3-month home-based intervention was able to improve functionality in post-bariatric patients^26^.

This study was not without limitations. First, qualitative research relies on the veracity and accuracy of the collected information. In addition, the analysis can be subjective to the researcher's interpretation, limited sample size, and only a female sample. Nonetheless, this study demonstrated great relevance in understanding the impact of the pandemic on the social aspects and mental health of patients who underwent bariatric surgery.

Finally, the use of technology was regarded as an innovative solution for health care through information and telemedicine, as well as a potential tool to become more physically active.

## CONCLUSIONS

The study showed a negative impact of the COVID-19 pandemic on aspects of mental and emotional health mostly due to lockdown measures, which led to social isolation and an increased burden with household chores. However, bariatric surgery was mentioned as a positive aspect of coping with clinical risk conditions. In addition, for some patients, the quarantine did provide the opportunity to spend more time with children, spouses, and OFMs.

Future studies should investigate whether the negative impacts of the pandemic on the mental health and well-being of this population persist over time, as well as possible therapies to lessen them.

Central Message Although social distancing measures were effective in preventing COVID-19 infection, mobility restrictions, lack of social interaction, and limitation of daily activities impacted individuals’ mental health, generating frustration, feelings of insecurity, confusion, emotional isolation, and stigma. Indeed, several quantitative studies have demonstrated various psychological symptoms arising from the COVID-19 quarantine, such as emotional imbalance, depression, stress, lack of mood, irritability, insomnia, post-traumatic stress symptoms, anger, and emotional exhaustion in working conditions. These effects have been particularly detrimental to people with obesity, with reports of increased anxiety and depression due to social isolation measures.

Perspectives The study showed a negative impact of the COVID-19 pandemic on aspects of mental and emotional health mostly due to lockdown measures, which led to social isolation and an increased burden with household chores. However, bariatric surgery was mentioned as a positive aspect of coping with clinical risk conditions. Additionally, for some patients, the quarantine did provide the opportunity to spend more time with children, spouses, and other family members.
